# Exercise Prescription and the Minimum Dose for Bone Remodeling Needed to Prevent Osteoporosis in Postmenopausal Women: A Systematic Review

**DOI:** 10.7759/cureus.25993

**Published:** 2022-06-16

**Authors:** Feeba Sam Koshy, Kitty George, Prakar Poudel, Roopa Chalasani, Mastiyage R Goonathilake, Sara Waqar, Sheeba George, Wilford Jean-Baptiste, Amina Yusuf Ali, Bithaiah Inyang, Lubna Mohammed

**Affiliations:** 1 Research, California Institute of Behavioral Neurosciences & Psychology, Fairfield, USA

**Keywords:** strength training, aquatic exercise, wbv, hiit, exercise, physical activity, bone mineral content, bone mineral density, postmenopausal bone loss, postmenopausal osteoporosis

## Abstract

The aim of this review is to analyze previously conducted randomized controlled trials and investigate the relationship between various exercise regimes and their effect on bone mineral density in postmenopausal women. To determine whether exercise can be used as a non-pharmacological modality for osteoporosis prevention, a thorough search was performed on various databases (PubMed, ScienceDirect, and Google Scholar). Only bone mineral density studies and trials with intervention versus control groups were included, and 13 randomized controlled trials were deemed relevant. The majority of trials concluded that exercise positively impacted bone mineral density in postmenopausal women. High-impact exercises seem to have the most significant effect on bone mineral density due to compression, shear stress, and high loading on the bone, causing bone remodeling. Considering all the limitations, exercise seems to be an effective tool for preventing postmenopausal osteoporosis.

## Introduction and background

“Osteoporosis is not an inevitable part of aging: it is preventable. So, it is vital that all of us, of all ages, start taking care of our bones now before it is too late” [1].

Osteoporosis is a major global health issue; its silent character results in despair due to loss of autonomy, chronic pain, disability, and increased morbidity and mortality [2]. Around 30% of all postmenopausal women in the United States and the European Union combined have osteoporosis, and 40% of them combined are predicted to suffer from one or more osteoporotic fractures during their lifetime [3]. Due to the increasing prevalence of osteoporosis after menopause, it is of paramount importance to prevent the progression of this disease before the damage advances to a point where it decreases function [2].

**Figure 1:**
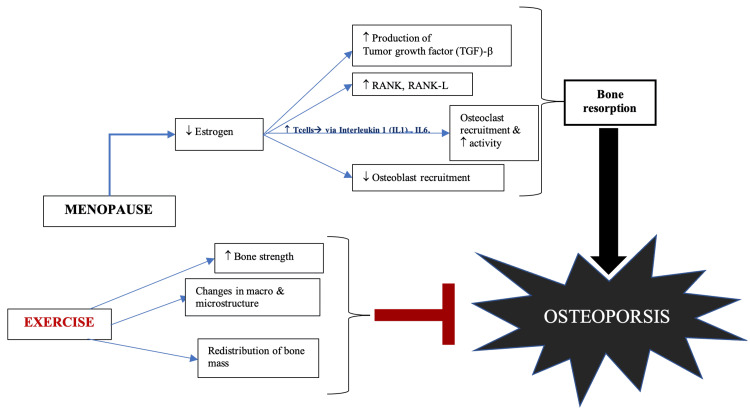
Pathophysiology of osteoporosis and the effects of exercise on the bone Down arrow: decreased or low; up arrow: increased or high RANK, receptor activator of NF-kB; RANK-L, receptor activator of NF-kB ligand

Clinical osteoporosis is defined as the loss of quality and integrity of the microstructure of the bone, decreased bone mineral density (BMD) (≤ -2.5 standard deviation [SD]), and therefore heightened fracture risk [4]. Osteopenia is associated with intermediate fracture risk where the value of BMD is between 1 and -2.5 SD below peak bone mass [5]. The etiology of osteoporosis can be attributed to many modifiable (social habits, physical activity, and diet) and non-modifiable (such as gender, age, and ethnicity) risk factors, which can contribute to bone loss either individually or synergistically [6]. One of the major contributing factors in the development of postmenopausal osteoporosis is estrogen deficiency [7]. Lack of estrogen increases osteoclast recruitment and decreases osteoblast production, thus causing an imbalance between bone resorption and bone formation, as shown in Figure 1 [8,9].

Despite the effectiveness of different pharmacological treatments, increased cases of poor long-term compliance and adverse drug effects [10] have led to exercise becoming an effective non‐pharmacological approach for maintaining bone health and preventing bone resorption, but with none of the side effects [11].

Exercise is known to reduce postmenopausal bone loss, specifically strength training, owing to increased mechanical loading. This leads to redistribution of bone mass and remodeling of its macro- and microstructure, thus maintaining or at the most improving BMD [12,13]. The very basis of bone strength is conditional on the collagen matrix and the degree of bone mineralization [6]. Previous studies report that a combination of weight-bearing and resistance exercises has increased the bone formation markers due to enhanced activity of the subperiosteal osteoblasts, thus strengthening the bone and decreasing susceptibility to fractures [14,15].

However, the effects of exercise on bone mass in postmenopausal women are met with scrutiny; some studies indicate that exercise could result in benefits [13,16], and some report no effects [17,18]. Other studies report a negative impact of exercise in postmenopausal women [18]. In one of the studies, exercise led to the loss of fat mass, thus exerting an influence on estradiol levels. As adipose tissue is the site of conversion from androgen to estrogen by aromatase, this led to a decrease in circulating estrogen, negatively impacting BMD [18].

This review will look at the different exercise modalities, such as aquatic-based exercise, resistance, strength, high-intensity interval training (HIIT), and whole-body vibration (WBV) exercise, and their effect on BMD or any other factors leading to the formation or breakdown of bone. This systematic review aims to summarize whether exercise is beneficial in preventing postmenopausal osteoporosis and if there is a minimum threshold that needs to be reached to elicit a structural change in the bone or is it just an intervention sitting on a glorified pedestal.

## Review

Methods

Preferred Reporting Items for Systematic Reviews and Meta-Analyses (PRISMA) 2020 guidelines were used to conduct and describe the findings of this systematic literature review [19]. Patient consent and ethical approval were not needed as this review required no patient contact or any influence on patient care. Any inconsistencies in the retrieval of articles or methodology were settled unanimously by all participating authors.

Search Strategy 

PubMed (Medline), ScienceDirect, and Google Scholar databases were used to conduct a thorough search of relevant studies and articles. Gray literature was also explored. The last date of all searches was on February 25, 2022. We used Medical Subject Heading (MeSH) terms and appropriate keywords to identify all pertinent articles relating to the “effect of exercise on preventing postmenopausal osteoporosis.” A combination of keywords such as “post-menopausal osteoporosis,” “postmenopausal bone loss,” “exercise,” “physical activity,” “bone mineral density,” and “prevention,” with the addition of MeSH terms and with the application of the Boolean method was used to synthesize a uniform search throughout the databases, as highlighted in Table 1.

**Table 1:** Bibliographic search strategy with the corresponding filters and results yielded (presented in alphabetic order)

Database	Search strategy	Filters	No. of results
PubMed (Medline)	Postmenopausal osteoporosis OR postmenopausal bone loss OR bone destruction OR ("Osteoporosis, Postmenopausal/physiopathology"[Majr] OR "Osteoporosis, Postmenopausal/prevention and control"[Majr] ) AND Exercise OR "Exercise/prevention and control"[Mesh]	Humans, English, female, middle aged: 45-64 years, aged: 65+ years, 80 and over: 80+ years. 2006-2022	441 results
ScienceDirect	Exercise AND Prevention of osteoporosis AND Postmenopausal women	2006-2022 Article type: research articles; subject area: medicine and dentistry	768 results
Google Scholar	allintitle: exercise in the prevention of postmenopausal osteoporosis	2006-2022; all article types	7 results

Inclusion and Exclusion Criteria 

Based on the abstract, studies were assessed for the potential to be included. The full article was included if they met the following criteria: (a) women-only studies; (b) studies including women who were not to be on any medications that altered bone metabolism other than recommended daily supplements such as calcium and/or vitamin D; (c) studies including women with postmenopausal status at study onset (cessation of menstrual periods for 12 consecutive months); (d) studies including women who were able to participate in physical activity; (e) studies including women who were healthy or only women diagnosed with osteoporosis/osteopenia with no other major comorbidities; and (f) randomized controlled trials (RCTs) with at least one exercise/training group and a comparative control group who participated in no exercise. Any studies with mixed genders or mixed pre- and postmenopausal status were excluded.

Data Selection and Extraction

**Figure 2:**
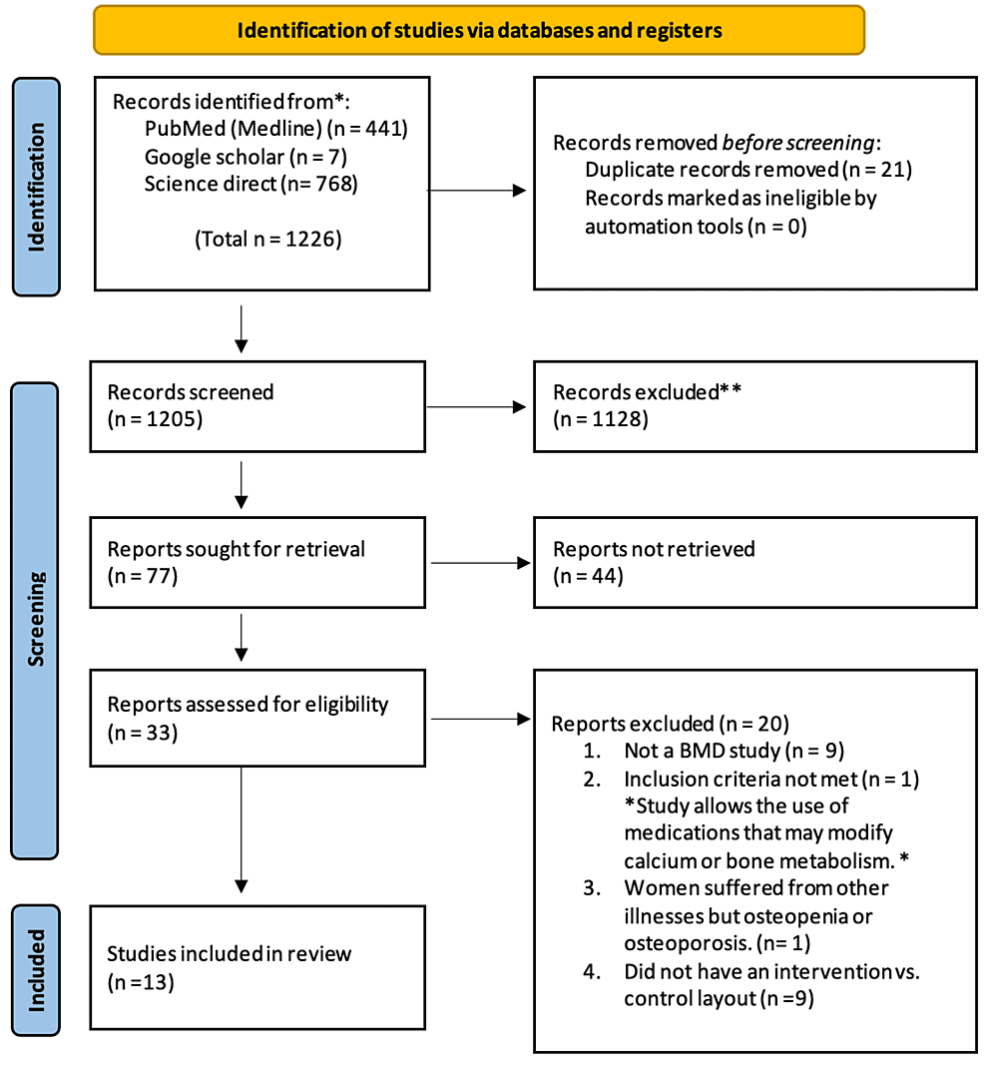
Flow diagram showing the process of study selection

We restricted our search to (a) studies and articles published between 2006 and 2022; (b) English-only articles; and (c) humans-only studies. Across all three databases, a total of 1,205 articles were identified after the removal of duplicate articles through the software Endnote. Two stages of screening then took place. Stage 1 screen was based on the titles and abstracts of the articles and removed any articles that we felt would not have contributed to this review. At stage 2 screening, we preceded to read full texts, and based on the eligibility criteria, reached through uniform consensus of both researchers; a total of 13 articles were chosen. The PRISMA flow diagram in Figure 2 highlights the steps taken in selecting full articles examined in this review.

Quality Assessment 

Two independent researchers evaluated each study for the potential risk of bias. The Cochrane Risk Bias Assessment Tool (RoB 2 tool) was used for RCTs comprised of five domains. The accepted articles were those with low risk or concern in only one domain, as shown in Table 2. All 13 RCTs were found to be of good quality. We attempted to minimize the risk of bias across studies by choosing articles with positive, negative, and no change results following a specific intervention. Despite this, there was plenty of variability between the studies, such as differences in sample size, diagnosis of patient condition, and interventions. Therefore, our primary aim was to focus on the outcomes regarding BMD.

**Table 2:** Risk bias: outcomes of ROB2 tool Y, yes; PY, probably yes; PN, probably no; N, no; NI, no information

Article	Domain 1	Domain 2	Domain 3	Domain 4	Domain 5	Risk of Bias judgment
Aboarrage Junior et al., 2018 [20]	1.1 Y/PY/PN/N/NI 1.2 Y/PY/PN/N/NI 1.3 Y/PY/PN/N/NI Low risk	2.1 Y/PY/PN/N/NI 2.2 Y/PY/PN/N/NI 2.3 Y/PY/PN/N/NI 2.4 Y/PY/PN/N/NI 2.5 Y/PY/PN/N/NI 2.6 Y/PY/PN/N/NI 2.7 Y/PY/PN/N/NI Low risk	3.1 Y/PY/PN/N/NI 3.2 Y/PY/PN/N/NI 3.3 Y/PY/PN/N/NI 3.4 Y/PY/PN/N/NI Low risk	4.1 Y/PY/PN/N/NI 4.2 Y/PY/PN/N/NI 4.3 Y/PY/PN/N/NI 4.4 Y/PY/PN/N/NI 4.5 Y/PY/PN/N/NI Low risk	5.1 Y/PY/PN/N/NI 5.2 Y/PY/PN/N/NI 5.3 Y/PY/PN/N/NI Low risk	Low risk
Basat et al., 2013 [21]	1.1 Y/PY/PN/N/NI 1.2 Y/PY/PN/N/NI 1.3 Y/PY/PN/N/NI Low risk	2.1 Y/PY/PN/N/NI 2.2 Y/PY/PN/N/NI 2.3 Y/PY/PN/N/NI 2.4 Y/PY/PN/N/NI 2.5 Y/PY/PN/N/NI 2.6 Y/PY/PN/N/NI 2.7 Y/PY/PN/N/NI Low risk	3.1 Y/PY/PN/N/NI 3.2 Y/PY/PN/N/NI 3.3 Y/PY/PN/N/NI 3.4 Y/PY/PN/N/NI Low risk	4.1 Y/PY/PN/N/NI 4.2 Y/PY/PN/N/NI 4.3 Y/PY/PN/N/NI 4.4 Y/PY/PN/N/NI 4.5 Y/PY/PN/N/NI Low risk	5.1 Y/PY/PN/N/NI 5.2 Y/PY/PN/N/NI 5.3 Y/PY/PN/N/NI Low risk	Low risk
Bocalini et al., 2009 [22]	1.1 Y/PY/PN/N/NI 1.2 Y/PY/PN/N/NI 1.3 Y/PY/PN/N/NI Low risk	2.1 Y/PY/PN/N/NI 2.2 Y/PY/PN/N/NI 2.3 Y/PY/PN/N/NI 2.4 Y/PY/PN/N/NI 2.5 Y/PY/PN/N/NI 2.6 Y/PY/PN/N/NI 2.7 Y/PY/PN/N/NI Low risk	3.1 Y/PY/PN/N/NI 3.2 Y/PY/PN/N/NI 3.3 Y/PY/PN/N/NI 3.4 Y/PY/PN/N/NI Low risk	4.1 Y/PY/PN/N/NI 4.2 Y/PY/PN/N/NI 4.3 Y/PY/PN/N/NI 4.4 Y/PY/PN/N/NI 4.5 Y/PY/PN/N/NI Low risk	5.1 Y/PY/PN/N/NI 5.2 Y/PY/PN/N/NI 5.3 Y/PY/PN/N/NI Low risk	Low risk
Chubak et al., 2006 [18]	1.1 Y/PY/PN/N/NI 1.2 Y/PY/PN/N/NI 1.3 Y/PY/PN/N/NI Low risk	2.1 Y/PY/PN/N/NI 2.2 Y/PY/PN/N/NI 2.3 Y/PY/PN/N/NI 2.4 Y/PY/PN/N/NI 2.5 Y/PY/PN/N/NI 2.6 Y/PY/PN/N/NI 2.7 Y/PY/PN/N/NI Low risk	3.1 Y/PY/PN/N/NI 3.2 Y/PY/PN/N/NI 3.3 Y/PY/PN/N/NI 3.4 Y/PY/PN/N/NI Low risk	4.1 Y/PY/PN/N/NI 4.2 Y/PY/PN/N/NI 4.3 Y/PY/PN/N/NI 4.4 Y/PY/PN/N/NI 4.5 Y/PY/PN/N/NI Low risk	5.1 Y/PY/PN/N/NI 5.2 Y/PY/PN/N/NI 5.3 Y/PY/PN/N/NI Low risk	Low risk
Engelke K et al. 2006 [23]	1.1 Y/PY/PN/N/NI 1.2 Y/PY/PN/N/NI 1.3 Y/PY/PN/N/NI Some concerns	2.1 Y/PY/PN/N/NI 2.2 Y/PY/PN/N/NI 2.3 Y/PY/PN/N/NI 2.4 Y/PY/PN/N/NI 2.5 Y/PY/PN/N/NI 2.6 Y/PY/PN/N/NI 2.7 Y/PY/PN/N/NI Low risk	3.1 Y/PY/PN/N/NI 3.2 Y/PY/PN/N/NI 3.3 Y/PY/PN/N/NI 3.4 Y/PY/PN/N/NI Low risk	4.1 Y/PY/PN/N/NI 4.2 Y/PY/PN/N/NI 4.3 Y/PY/PN/N/NI 4.4 Y/PY/PN/N/NI 4.5 Y/PY/PN/N/NI Low risk	5.1 Y/PY/PN/N/NI 5.2 Y/PY/PN/N/NI 5.3 Y/PY/PN/N/NI Low risk	Some concerns
Englund et al., 2009 [24]	1.1 Y/PY/PN/N/NI 1.2 Y/PY/PN/N/NI 1.3 Y/PY/PN/N/NI Low risk	2.1 Y/PY/PN/N/NI 2.2 Y/PY/PN/N/NI 2.3 Y/PY/PN/N/NI 2.4 Y/PY/PN/N/NI 2.5 Y/PY/PN/N/NI 2.6 Y/PY/PN/N/NI 2.7 Y/PY/PN/N/NI Low risk	3.1 Y/PY/PN/N/NI 3.2 Y/PY/PN/N/NI 3.3 Y/PY/PN/N/NI 3.4 Y/PY/PN/N/NI Low risk	4.1 Y/PY/PN/N/NI 4.2 Y/PY/PN/N/NI 4.3 Y/PY/PN/N/NI 4.4 Y/PY/PN/N/NI 4.5 Y/PY/PN/N/NI Low risk	5.1 Y/PY/PN/N/NI 5.2 Y/PY/PN/N/NI 5.3 Y/PY/PN/N/NI Low risk	Low risk
Kemmler et al., 2015 [25]	1.1 Y/PY/PN/N/NI 1.2 Y/PY/PN/N/NI 1.3 Y/PY/PN/N/NI Some concerns	2.1 Y/PY/PN/N/NI 2.2 Y/PY/PN/N/NI 2.3 Y/PY/PN/N/NI 2.4 Y/PY/PN/N/NI 2.5 Y/PY/PN/N/NI 2.6 Y/PY/PN/N/NI 2.7 Y/PY/PN/N/NI Low risk	3.1 Y/PY/PN/N/NI 3.2 Y/PY/PN/N/NI 3.3 Y/PY/PN/N/NI 3.4 Y/PY/PN/N/NI Low risk	4.1 Y/PY/PN/N/NI 4.2 Y/PY/PN/N/NI 4.3 Y/PY/PN/N/NI 4.4 Y/PY/PN/N/NI 4.5 Y/PY/PN/N/NI Low risk	5.1 Y/PY/PN/N/NI 5.2 Y/PY/PN/N/NI 5.3 Y/PY/PN/N/NI Low risk	Some concerns
Lai et al. 2013 [26]	1.1 Y/PY/PN/N/NI 1.2 Y/PY/PN/N/NI 1.3 Y/PY/PN/N/NI Low risk	2.1 Y/PY/PN/N/NI 2.2 Y/PY/PN/N/NI 2.3 Y/PY/PN/N/NI 2.4 Y/PY/PN/N/NI 2.5 Y/PY/PN/N/NI 2.6 Y/PY/PN/N/NI 2.7 Y/PY/PN/N/NI Low risk	3.1 Y/PY/PN/N/NI 3.2 Y/PY/PN/N/NI 3.3 Y/PY/PN/N/NI 3.4 Y/PY/PN/N/NI Low risk	4.1 Y/PY/PN/N/NI 4.2 Y/PY/PN/N/NI 4.3 Y/PY/PN/N/NI 4.4 Y/PY/PN/N/NI 4.5 Y/PY/PN/N/NI Low risk	5.1 Y/PY/PN/N/NI 5.2 Y/PY/PN/N/NI 5.3 Y/PY/PN/N/NI Low risk	Low risk
Montgomery et al., 2020 [17]	1.1 Y/PY/PN/N/NI 1.2 Y/PY/PN/N/NI 1.3 Y/PY/PN/N/NI Low risk	2.1 Y/PY/PN/N/NI 2.2 Y/PY/PN/N/NI 2.3 Y/PY/PN/N/NI 2.4 Y/PY/PN/N/NI 2.5 Y/PY/PN/N/NI 2.6 Y/PY/PN/N/NI 2.7 Y/PY/PN/N/NI Low risk	3.1 Y/PY/PN/N/NI 3.2 Y/PY/PN/N/NI 3.3 Y/PY/PN/N/NI 3.4 Y/PY/PN/N/NI Low risk	4.1 Y/PY/PN/N/NI 4.2 Y/PY/PN/N/NI 4.3 Y/PY/PN/N/NI 4.4 Y/PY/PN/N/NI 4.5 Y/PY/PN/N/NI Low risk	5.1 Y/PY/PN/N/NI 5.2 Y/PY/PN/N/NI 5.3 Y/PY/PN/N/NI Low risk	Low risk
Nicholson et al., 2015 [27]	1.1 Y/PY/PN/N/NI 1.2 Y/PY/PN/N/NI 1.3 Y/PY/PN/N/NI Low risk	2.1 Y/PY/PN/N/NI 2.2 Y/PY/PN/N/NI 2.3 Y/PY/PN/N/NI 2.4 Y/PY/PN/N/NI 2.5 Y/PY/PN/N/NI 2.6 Y/PY/PN/N/NI 2.7 Y/PY/PN/N/NI Low risk	3.1 Y/PY/PN/N/NI 3.2 Y/PY/PN/N/NI 3.3 Y/PY/PN/N/NI 3.4 Y/PY/PN/N/NI Low risk	4.1 Y/PY/PN/N/NI 4.2 Y/PY/PN/N/NI 4.3 Y/PY/PN/N/NI 4.4 Y/PY/PN/N/NI 4.5 Y/PY/PN/N/NI Low risk	5.1 Y/PY/PN/N/NI 5.2 Y/PY/PN/N/NI 5.3 Y/PY/PN/N/NI Low risk	Low risk
Sen et al. 2020 [28]	1.1 Y/PY/PN/N/NI 1.2 Y/PY/PN/N/NI 1.3 Y/PY/PN/N/NI Low risk	2.1 Y/PY/PN/N/NI 2.2 Y/PY/PN/N/NI 2.3 Y/PY/PN/N/NI 2.4 Y/PY/PN/N/NI 2.5 Y/PY/PN/N/NI 2.6 Y/PY/PN/N/NI 2.7 Y/PY/PN/N/NI Low risk	3.1 Y/PY/PN/N/NI 3.2 Y/PY/PN/N/NI 3.3 Y/PY/PN/N/NI 3.4 Y/PY/PN/N/NI Low risk	4.1 Y/PY/PN/N/NI 4.2 Y/PY/PN/N/NI 4.3 Y/PY/PN/N/NI 4.4 Y/PY/PN/N/NI 4.5 Y/PY/PN/N/NI Low risk	5.1 Y/PY/PN/N/NI 5.2 Y/PY/PN/N/NI 5.3 Y/PY/PN/N/NI Low risk	Low risk
Von Stengel et al., 2011 [29]	1.1 Y/PY/PN/N/NI 1.2 Y/PY/PN/N/NI 1.3 Y/PY/PN/N/NI Low risk	2.1 Y/PY/PN/N/NI 2.2 Y/PY/PN/N/NI 2.3 Y/PY/PN/N/NI 2.4 Y/PY/PN/N/NI 2.5 Y/PY/PN/N/NI 2.6 Y/PY/PN/N/NI 2.7 Y/PY/PN/N/NI Low risk	3.1 Y/PY/PN/N/NI 3.2 Y/PY/PN/N/NI 3.3 Y/PY/PN/N/NI 3.4 Y/PY/PN/N/NI Low risk	4.1 Y/PY/PN/N/NI 4.2 Y/PY/PN/N/NI 4.3 Y/PY/PN/N/NI 4.4 Y/PY/PN/N/NI 4.5 Y/PY/PN/N/NI Low risk	5.1 Y/PY/PN/N/NI 5.2 Y/PY/PN/N/NI 5.3 Y/PY/PN/N/NI Low risk	Low risk
Wochna et al., 2019 [30]	1.1 Y/PY/PN/N/NI 1.2 Y/PY/PN/N/NI 1.3 Y/PY/PN/N/NI Some concerns	2.1 Y/PY/PN/N/NI 2.2 Y/PY/PN/N/NI 2.3 Y/PY/PN/N/NI 2.4 Y/PY/PN/N/NI 2.5 Y/PY/PN/N/NI 2.6 Y/PY/PN/N/NI 2.7 Y/PY/PN/N/NI Low risk	3.1 Y/PY/PN/N/NI 3.2 Y/PY/PN/N/NI 3.3 Y/PY/PN/N/NI 3.4 Y/PY/PN/N/NI Low risk	4.1 Y/PY/PN/N/NI 4.2 Y/PY/PN/N/NI 4.3 Y/PY/PN/N/NI 4.4 Y/PY/PN/N/NI 4.5 Y/PY/PN/N/NI Low risk	5.1 Y/PY/PN/N/NI 5.2 Y/PY/PN/N/NI 5.3 Y/PY/PN/N/NI Low risk	Some concerns

Characteristics of Articles Collected 

Out of the 13 collected articles, all of them are RCTs. Table 3 below highlights the design and goals of each study.

**Table 3:** Aim of the study (presented in alphabetical order) BMC, bone mineral content; BMD, bone mineral density; CMJ, countermovement jump; HIIAE, high-intensity jump-based aquatic exercise program; HRQoL, health-related quality of life; WBV, whole-body vibration

	Author, year	Intervention duration	Aim of the study
1	Aboarrage Junioret al,. 2018 [20]	Duration: 24 weeks, three times a week	How bone mass and functional fitness are affected following an HIIAE program in postmenopausal women
2	Basat et al,. 2013 [21]	Duration: six months, three times a week	The impact strengthening and high-impact exercise training has on BMD, bone turnover markers, and HRQoL in postmenopausal women
3	Bocalini et al., 2009 [22]	Duration: 24 weeks, three times a week	How BMD is affected following strength training in postmenopausal women without hormone replacement therapy
4	Chubak et al., 2006 [18]	Duration: 12 months, five times a week	The effects physical activity has on BMD, BMC, and lean mass in postmenopausal, overweight/obese women
5	Engelke et al., 2006 [23]	Duration: three years, two group sessions per week (60-70 mins each) + two home sessions per week (25 mins each)	How to cease or slow bone loss during the early postmenopausal years
6	Englund et al., 2009 [24]	Duration: 12 months, two times a week + five weeks break + five-year follow-up	To see if BMD and neuromuscular function gains made during weight-bearing program are lost after a long period of exercise cessation
7	Kemmler et al., 2015 [25]	Duration: results of a 16-year trial, 49-50 weeks per year	To see changes in clinical overall fracture incidence and BMD in elderly subjects following exercise
8	Lai et al., 2013 [26]	Duration: six months, three times a week	How LS BMD is affected following high-frequency and high-magnitude WBV in postmenopausal women
9	Montgomery et al., 2020 [17]	Duration: 12 months, three times a week	To evaluate if continuous and intermittent CMJ intervention reduces early postmenopausal BMD losses
10	Nicholson et al., 2015 [27]	Duration: six months, two times a week	How BMD and body composition is affected by six months of low-load, very high repetition resistance training in non-osteoporotic women
11	Sen et al., 2020 [28]	Duration: 34 weeks, three times a week	To evaluate the effects of WBV and high-impact exercises on postmenopausal women
12	Von Stengel et al., 2011 [29]	Duration: 18 months two times a week	To see how WBV influences BMD and falls.
13	Wochna et al., 2019 [30]	Duration: six months, two times a week	How aqua fitness training in deep water affects bone tissue

Results

Across all three databases, a total of 1,205 articles were identified after the removal of duplicate articles through the software Endnote. At the first stage of screening, 1,128 articles that we felt would not have contributed to this review were removed. After stage 2 screening, we ended up with a total of 13 articles. The study, intervention, and conclusions (effect on BMD) are presented in Table 4.

**Table 4:** Intervention type and conclusions of selected studies (Presented in alphabetical order). 1RM, one-repetition maximum; BMC, bone mineral content; BMD, bone mineral density; BMI, body max index; CG, control group; CMJ- INT, intermittent countermovement jumps; CMJ-CTS, continuous countermovement jumps; DXA, dual-energy X-ray absorptiometry; EG, exercise group; FN, femoral neck; HG, high-impact training group; HIIT, high-intensity interval training; LS, lumbar spine; mins, minutes; NTx, N-telopeptides of type 1 collagen; OC, osteocalcin; TG, training group; TGV, training group + vibration; TR, trained; UN, untrained group; WBV, whole-body vibration *Body- Pump^TM^ training group which is a pre-choreographed group class that uses light weights and very high (80–100) repetitions

	Study	Participants	Intervention	Conclusion	
1	Aboarrage Junior et al., 2018 [20]	Age range: 65 ± 7 years	Type of training: aquatic training session	Significant differences were found in the BMD of the hip, LS, and whole body of the T group when compared with the UN group. The data from this study suggest that aquatic-based exercise can improve BMD and functional fitness in postmenopausal women	
			Stages: 5 mins warm-up, 20 mins jump-based exercise performed as HIIT: 20 reps for 30 secs and 5 mins cooling down		
		Study groups and distribution: TG, n = 15; UN, n = 10; total n = 25			
2	Basat et al,. 2013 [21]	Women with osteopenia (BMD at LS and/or FN between −1.0 and −2.5); age range: strength training = 55.9 ± 4.9 years; high impact = 55.6 ± 2.9 years; CG = 56.2 ± 4.0 years	Type of training: strength or high intensity	BMD of the LS and FN increased in both strength training and high-intensity TGs; however, there was a decrease in BMD in the CG. A significant increase in serum OC was seen in both TGs and a nonsignificant increase in the CG. N-telopeptides of type 1 collagen (NTx) levels were increased in the CG; however, it was decreased in both TGs. This study concluded that high-impact exercise training can be effective in the prevention of bone loss at the level of the LS and FN.	
			Stages: warm-up period (bicycling, walking in place, static stretching exercises), strengthening exercises or high-impact exercises (jump rope), cooldown period		
		Study groups and distribution: strength training, n = 14; high impact, n = 14; CG, n = 14; total n = 42			
3	Bocalini et al., 2009 [22]	Age range: TR = 69 ± 9; UN = 67 ± 8	Type of training: strength training program	TR group showed no significant demineralization in the LS or FN, whereas the UN group had a substantial decrease in BMD of the LS and FN. Body composition parameters (BMI and body fat %) were lower in the TR group than in the UN group. The data from this study showed improved body composition parameters and preserved BMD in postmenopausal women.	
			Stages: 10 min warm- up one set at 50% of the one repetition maximum load (1RM) 3 sets of 10 repetitions for given exercise at 85% of 1RM. Types of strength exercises performed include: Leg press, chest press, leg curl, latissimus pull down, elbow flexion, elbow extension, leg extension, upper back row, military press, hip abductor, hip adductor, and abdominal curls		
		Study groups and distribution: TR, n = 23; UN, n = 12; total n = 35			
4	Chubak et al., 2006 [18]	Women without known osteoporosis or osteopenia. Age range: exercisers = 60.6 ± 6.8; stretchers (CG) = 60.7 ± 6.7	Type of training: moderate-intensity aerobic training exercise	TG had no significant changes in BMD, BMC. Exercisers lost more weight than stretchers in the 12-month period. Conclusion: this study concluded that there were no significant changes in BMD, BMC, and body fat in both exercises and stretchers.	
			Stages: 40% of observed maximal heart rate for 16 mins per session; increase to 60-70% of maximal heart rate for 45 mins per session. Type of training: moderate-intensity aerobic training exercise (walking and bicycling)		
		Study groups and distribution: exercisers (TG), n = 87; stretchers (CG), n = 86; total n = 173			
5	Engelke et al., 2006 [23]	Women with osteopenia (BMD of LS or total proximal femur )1> DXA T- score >)2.5 SD) Age range of patients included at 3-year analysis: EG = 55.1 ± 3.3; CG = 55.5 ± 3.0	Type of training: low-volume high-resistance strength training and high-impact aerobics	Within the EG, there were positive LS BMD changes; however, in the CG LS, BMD was significantly decreased. The proximal femur BMD was maintained in EG, whereas femur BMD in CG was significantly reduced. Forearm BMD for both groups was decreased significantly. This three-year study was successful in maintaining BMD at the level of the spine and hip but not at the forearm.	
			Stages: warm-up: gradually increased walking and running program in the first 3 months. Jumping sequence: started after 6 months. Strength- training sequence. Flexibility training sequence. Home sessions were 20-25 mins; every 12 weeks the intensity was increased.		
		Study groups and distribution: At baseline: EG, n = 86 CG, n = 51 total n = 137. Included in three-year analysis: EG = 48; CG = 30; total n = 78			
6	Englund et al., 2009 [24]	Age range of patients included at 5-year follow-up: EG = 55.1 ± 3.3 CG = 55.5 ± 3.0	Type of training: Combination of strength, aerobic, balance, and coordination training.	EG showed significant increases in BMD from baseline compared to CG. However, both groups had losses of BMC at the FN, trochanter between the end of trial and the 5-year follow-up visit. Three participants continued to exercise in the follow-up period and results showed preservation of their neuromuscular parameters. This study concluded that any BMD gains made during exercise are lost if the exercise regime is stopped for a long period of time.	
		Study groups and distribution: at baseline: EG, n = 86; CG, n = 51; total n = 137. Included in the 5-year follow-up: EG = 18; CG = 16; total n = 34			
7	Kemmler et al., 2015 [25]	Women with osteopenia. Age range of patients included at 3-year analysis: EG = 55.3 ± 3.4; CG = 55.5 ± 3.2	Type of training: multipurpose exercise program	Both groups showcased decreased BMD but the reduction was greater in the CG. They concluded the study showed high anti-fracture efficiency as a result of exercise.	
			Stages: Two group classes = 60-65 mins. 5-10 min running/dancing. 10-15 mins of low- and high-impact (4 sets 15 reps of multiple jumping exercises) aerobic dance exercise with peak ground reaction forces at 2-3 times above body weight. Resistance exercises on machines. Two home training sessions = 20-25 mins		
		Study groups and distribution: at baseline: EG, n = 86; CG, n = 51; total n = 137. Included in 16-year follow-up: EG = 59; CG = 46; total n = 105			
8	Lai et al., 2013 [26]	100% of women in WBV group had osteopenia or osteoporosis and 85% in the CG Age range: WBV training = 60.1 ± 7.1; UN = 62.4 ± 7.1	Type of training: WBV training. Subjects stood on a vibration device with a frequency of 30 Hz and a magnitude of 3.2 g for 5 mins each round.	There was an increase in BMD of the LS in the WBV group and a decrease in the CG, both were significant changes.	
		Study groups and distribution: WBV training, n = 14; CG, n = 14; total n = 28			
9	Montgomery et al., 2020 [17]	Age range: 54.6± 3.4	Type of training: CMJ-CTS and CMJ-INT were performed barefoot and told to “jump as high as possible”; CTS = 30 CMJs at a frequency of 15 jumps/min; INT = 30 CMJs at frequency of 4 jumps/min	When compared to all the groups, CG had the most significant reduction in LS and FN BMD. There was no significant difference in BMD of either LS or FN between the two intervention groups. The CG experience of BMD loss was almost three times higher than the intervention groups.	
		Study groups and distribution: CMJ-CTS, n = 9; CMJ-INT, n = 8; CG, n = 11; total n = 28			
10	Nicholson et al., 2015 [27]	Healthy women. Age range: 54.6± 3.4. Intervention group (PUMP*): 66± 4.4; CG = 66 ±4.5	Type of training: low-load very high-repetition resistance training	PUMP group showed insignificant LS BMD increase and significant total body BMD decrease. CG showed a significant decrease of LS BMD and nonsignificant change for total body BMD.	
			Stages: warm up, 2 BodyPumpTM classes per week which work on the legs chest, back, triceps, biceps, lunges, shoulders and core, cool down		
		Study groups and distribution: PUMP, n = 24; CG, n = 26; total n = 50			
11	Sen et al., 2020 [28]	Women with osteoporosis (BMD T scores between -2.0 and -3.0). Age range at baseline: WBV training = 55.0 ± 4.6; HG = 53.1 ± 4.4; CG = 54.5 ± 6.0	Type of exercises: WBV or high-intensity exercises	There was a significant increase in LS and FN BMD in the WBV compared to the CG. There was no change in BMD between the CG and HG. There was a significant decrease in serum OC levels in the WBV group compared to the other two groups.	
			Stages: 20-40 mins initial training program; warm-up (cycling, stepping), stretching, and strengthening exercises; WBV exercises (high frequency, 30-40Hz, in 5 different positions) or high-impact exercises (jump rope), cooldown		
		Study groups and distribution at baseline: WBV, n = 19; HG = 19; CG = 20; total n = 58			
12	Von Stengel et al., 2011 [29]	Age range at baseline: conventional TG = 68.6 ± 3.0; conventional TGV = 68.8 ± 3.6; CG = 68.1 ± 2.7	Type of exercise: high impact and vibration training	Both TG and TGV groups showed a significant increase in LS BMD, while no change occurred in the CG. The application of vibration does not enhance these effects.	
			Stages: TG: 20 mins dancing aerobics, 5 min balance training, 20 mins functional gymnastics, 15 mins dynamic leg-strength training on vibration plates (without vibration); TG with vibration: 20 mins dancing aerobics, 5 mins balance training, 20 mins functional gymnastics, 15 mins dynamic leg-strength training on vibration plates (25- 35Hz vibration)		
		Study groups and distribution at baseline: TG, n = 50; TGV = 50; CG = 51; total n = 151			
13	Wochna et al., 2019 [30]	Healthy women. Age range at baseline: TG = 58 ± 3.27; CG = 60 ± 3.37	Type of training: aqua fitness. The exercises took place in deep water with equipment such as pool noodles, water dumbbells, gloves, balls, and resistance bands	This study showed no significant changes in BMD values between the two groups. This study concluded that aqua training has a positive impact on femur strength index but had no changes in BMD.	
		Study groups and distribution at baseline: TG, n = 9; CG, n = 9; total n = 18			

Discussion

Bone health and aging are two concerns that commonly walk hand in hand. Aging is associated with bone demineralization, most commonly after the third or fourth decade [27], which can lead to spontaneous fractures, especially in women with estrogen deficiency [22].

Various strategies have and are being explored to negate the structural degradation of bone tissue. Physical activity has been considered a low-risk, low-cost treatment preference and is continually becoming a popular option. Maintaining bone density or preventing bone loss, in turn, helps reduce falls and fractures, improving patients’ health-related quality of life (HRQoL) [21,28]. The exercises of the reviewed studies are widely variable and heterogenous, ranging from aquatic programs to resistance regimes. The main primary purpose of this systematic review is to have a deeper understanding of the different training modalities, their effect on BMD, and their efficacy in preventing osteoporosis, as well as to decipher which exercise regime has the most significant impact on BMD and therefore the greatest degree of preventive effect on osteoporosis in postmenopausal women. Several studies established that exercise had a positive effect on BMD [20,21,23,26-29].

Some studies showed maintenance or lower decreases in BMD compared to control groups [17,22,24,25], and some concluded that no changes were seen in BMD following the intervention [18,30].

Aquatic Training

Studies show that water pressure at varying depths stimulates bone through muscle loading. An aquatic program also has the benefit of lower traumatic fracture risk instead of land-based exercises. The following two RCTs have conflicting results on the effect of aquatic activities on BMD [20,30].

The bulk of Aboarrage et al.’s study included jump-based activities in water, fixed at or near the xiphoid process. Movements such as single-leg jumps, ankle hops, and tuck jumps were performed at high intensity. Significant BMD increases were seen after the 24-week exercise protocol at the hip (before: 0.860 -0.070 vs. after: 1.040 -0.100), lumbar spine (LS) (before: 1.050 -0.016 vs. after: 1.090 -0.015), and whole body (before: 1.000 ± 0.011 vs. after: 1.060 ± 0.009), when compared to the control group. It was concluded that this study’s jump-based aquatic exercises resulted in increased BMD. The activities performed are thought to load joints and bone tissue, due to the biochemical reactions in bone cells, on the condition that the stimulus exceeds a certain threshold above the day-to-day standard to which the body is already adapted to [20].

On the contrary, Wochna et al.’s study concluded no significant changes in LS, total femur, femoral neck (FN), and total body BMD between the training and control groups after six months. The participants adhered to a 45-minute aquatic fitness class biweekly. The training included deep water (up to neckline) training involving water-friendly equipment such as pool noodles, dumbbells, gloves, balls, and resistance bands to increase the total body-to-water surface area. A significant increase in the femur strength index of the training group was seen. This was attributed to the resistance provided by the increased surface contact. The authors of this study owe this result to limitations in sample size and method of the conducted research [30].

Whole-Body Vibration Training

WBV has increasingly become an attractive option, especially for patients who cannot tolerate weight-bearing exercise prescriptions, such as those with joint, muscle, and nerve diseases [26,28,29]. It is an exercise where subjects stand straight on a platform, and the stimulation source transmits vibration vertically through the body [26,29]. WBV is shown to cause direct bone growth and stimulation by modifying bone fluid flow.

WBV can lead to adverse effects if individuals are exposed for an extended time or increased intensities. Therefore, the intensity and duration of the vibration are essential factors to account for to prevent side effects such as dizziness and headaches [26].

Lai et al.’s study used a high-frequency, high-magnitude WBV where participants were exposed to vibration with a frequency of 30Hz and a magnitude of 3.2g for five minutes at a time [26]. Ekin et al.’s study also used a similar vibration frequency of 35Hz with two sets totaling 5 minutes and an amplitude of 2mm, working up to 4mm by the end of the program [28]. Von Stengel et al.’s study started with an amplitude of 1.7mm and a slightly lower frequency of 25Hz, progressively increasing to 35Hz for 6 minutes per session [29]. In the current research, significant increases in LS BMD (2.032% ± 3.332%) were seen in the WBV group and a decrease in the control group (0.046% ± 1.245%) [26]. Moreover, another high-frequency but low-amplitude study conducted in 2020 shows a significant increase in LS (+1.3%) and FN (+5.0%) after six months in the WBV training group compared to the control group [28].

A study compared the effect of the training group, training group with the addition of WBV, and the control group on BMD. Despite the positive impact of LS BMD in both training groups, it was concluded that WBV did not lead to any further increases in BMD in addition to changes in BMD already identified with the conventional training group. However, WBV was shown to decrease the number of falls significantly. Due to sparse knowledge in this region, the reason for this result is unknown [29]. There is a possibility that this is due to improved functional mobility in postmenopausal women. Other evidence shows that WBV leads to the improvement of balance and postural control [28].

Resistance Training

High-impact resistance training with jumping sequences is shown to positively impact the bone due to their high strain properties, leading to increased muscular demand and a positive osteogenic effect on the recruited bony segments [17]. Engelke et al.’s study demonstrated the impact of a three-year program of integrated endurance, jumping, and high-intensity resistance training on BMD. In this study, LS and proximal femur BMD was maintained in the training group, and the control group continued having losses [23]. However, forearm BMD suffered a negative impact, likely explained by the lack of adequate skeletal compression and loading of the upper extremities [23].

Nicholson et al.’s study used another variation of resistance exercise as an intervention [27]. Before their study, the effects of low-load, high-repetition resistance training on BMD were never explored [27]. The study reported no significant changes in LS BMD in the training group. However, a substantial decrease in LS BMD was seen in the control group [27]. No other changes at any other sites were observed. The total body BMD was decreased in the training group [27]. The authors of this study hypothesize that low calcium intake and the absence of progressive overload played a part in the lack of improvement or maintenance of BMD. Due to the generality of the program mentioned in this study, there was a scarcity of skeletal compression and intense muscle contractions needed for the bone to undergo biochemical changes [27].

Strength and High-Impact Training

Strength and high-impact training is known to stimulate bone formation through mechanical loading coupled with high levels of muscular force. This theory is put to the test in the studies by Montgomery et al. [17], Basat et al. [21], Bocalini et al. [22], Englund et al. [24], Kemmler et al. [25], and Von Stengel et al. [29]. A study that took place in Istanbul reported an increase in LS and FN BMD following strength training (+1.3%, +1.6%) compared to high-impact exercise (+0.5%, +1.2%) respectively, but bone loss ensued in the control group (-2.5%, -1.0%) [21]. However, compared to the control group, high-impact training exhibited statistically significant changes in BMD than strength training [21]. This result is because of jumping, leading to increased loading of the femoral and hip joint, proving the proposed Wolff’s law, which states that the higher the degree of mechanical loading, the higher the degree of adaptation and changing of bone architecture, thus the formation of stronger bones [21].

Two other studies also concluded that strength and high-impact training led to significant increases in BMD [24,29]. However, any gains in BMD made during the intervention period were lost when the intervention was discontinued. The training and control groups suffered BMD losses at the five-year follow-up [24].

Three participants who continued physical activity in this study, who attended at least two scheduled group classes, showed no BMD losses, indicating that exercise does maintain BMD [24]. If not increased mineralization, some studies show maintenance of BMD following strength training [22]. There was a significant decrease in LS and FN BMD in the control group and preservation of BMD for both sites in the training group. This result is justified through the piezoelectric effect. This effect explains the balance between bone regeneration and bone degradation. Strength training induces mechanical stress on the bone causing bone collagen to slide past each other generating current, stimulating signaling pathways, opening voltage-gated calcium channels, and ultimately promoting bone formation [22].

Montgomery et al.’s study experimented with independent frequencies of mechanical loading and its effect on BMD [17]. Complex adaptive phenomenon hypothesis that rest intervals between given stimulus will allow maximal transient fluid flow within the bone system leading to a higher degree of responsiveness of the bone, thus enhancing its osteogenic potential, a theory tested in the study by Montgomery et al. [17]. Participants in the intervention group performed high-impact exercises of two different frequencies: continuous or intermittent. The results showed LS and FN BMD maintenance in both intervention and control groups experiencing significant losses in both LS and FN BMD. However, no significant differences in BMD were noted between the intervention groups [17].

However, a 16-year study with a multicomponent exercise regime as its intervention showed conflicting results [25]. The exercise program consisted of an endurance sequence (running/dancing), a high-impact sequence (jumping), and resistance exercises. LS and FN BMD were significantly reduced in both the training and control groups; however, the control group suffered a more marked decrease [25]. Nonetheless, using structured questionnaires and interviews, the overall fractures were calculated. It was concluded that the control group sustained more low-trauma fractures than the exercise group, verifying their initial hypothesis that multicomponent exercise program reduces fracture risk in postmenopausal women [25].

Other Types of Training

The subsequent RCT concluded that moderate-intensity aerobic exercise had no impact on bone structure [18]. Over 95% of activities were walking and bicycling. Not only this, but exercisers lost more body fat and weight compared to the control group. This leads to the theory that the benefit of exercise can be counteracted by the loss of adipose tissue, which decreases total estradiol concentrations, thus negating any potential BMD gains. It was observed in this study that women in the intervention group had a decrease in body fat and estradiol concentrations [18]. Due to the lack of knowledge in this area and fewer studies undertaken analyzing estradiol levels in individuals who exercise, it is unsure if there is a valid relationship [18].

Effect of Exercise on Other Parameters

Exercise influences BMD and as well as other parameters, such as functional fitness, used for the assessment of mobility and balance, especially in older adults [20,24,28]. Other parameters include but are not limited to HRQoL [21,28], bone mineral content [24], low-trauma fracture risk [25], fall risk [29], femur strength index [30], and body composition parameters (BMI, body fat %) [17,18,20-30] and bone turnover markers (serum osteocalcin [OC] and C-terminal telopeptide of type I collagen level) [28,30]. These markers exhibit bone resorption and bone formation. It is theorized that bone under stress will recruit osteocytes to form new bone, and once the stress falls below the threshold, this process is stopped [21]. This theory was disproven in two studies that showed decreased OC levels in the intervention group [28,30]. Decreased OC could be due to the overwhelming effect of menopause and short intervention periods.

Is There a Minimum Threshold Needed to Induce BMD Changes?

We decided to analyze articles with the shortest intervention period, those between 24 and 26 weeks, to answer this question. We perceived that women diagnosed with osteopenia made more significant and faster increases in BMD in a shorter period [21,26,28] than those who were healthy [27,30].

Both Aboarrage et al.’s and Bocalini et al.’s studies do not mention the status of the participant’s diagnosis at the start of the intervention [20,22]. They are therefore not included in answering this question.

The intervention period in the Basat et al. was 26 weeks, carried out three times a week [21]. The intervention included strengthening exercises performed for one set and ten repetitions per exercise for 30 minutes and high-impact exercises (jump rope) consisting of 10 jumps/day, + five jumps/week, and a maximum of 50 jumps/day for 10 minutes. There was a significant increase in LS BMD (+1.3%) and FN BMD (+1.6%) for the strength training group. The high-impact group also had a significant increase in BMD but not as high as the strength training group (LS BMD: +0.5%, FN BMD: +1.2%) [21]. We can see a higher degree of change in the studies that included WBV training [26,28]. In Lai et al.’s study, the intervention period and frequency were the same as in Basat et al.’s study [21,26]. The frequency and magnitude of the WBV were 30Hz and 3.2g for 5 minutes per session, respectively. There was a significant increase in LS BMD of +2.032% ± 3.332% [26]. Sen et al.’s study, despite the intervention period being less than all the studies, showcased the most significant degree of change in BMD [28]. The vibration frequency started at 30Hz (1 set/30 seconds), increasing to 35Hz (2 sets/5 minutes each) over 24 weeks [28]. They also included a high-intensity (jump rope) group, like Basat et al.’s study. The WBV groups’ LS BMD increased by +1.3% and the FN BMD by +5.0%, whereas there were no changes in BMD compared to the control group [28].

Due to differences in exercise modalities, it is not possible to say if one holds more precedence over the other based on my methods. However, we can see that between groups that had the addition of WBV, the group that increased frequency in increments led to a higher percentage of change in FN BMD. Studies with a shorter duration of intervention time should be undertaken to see if significant changes come to light indeed. Therefore, this systematic review has failed to uncover the minimum threshold for structural bone change.

Limitations

Across the studies, there are differences in the choice of the anatomic parts when measuring BMD. There were studies where some participants took vitamin D or calcium supplements while others did not within the same study, which could have possibly skewed the results. There was a lack of uniformity between intervention groups in the different control trials; therefore, there were fewer data to extrapolate valid results. Environmental factors such as smoking, alcohol use, and diet should be given importance when conducting future trials.

## Conclusions

The compilation of articles reviewed, in the majority, demonstrated that physical activity has improved or at the very least maintained BMD in postmenopausal women. The detrimental effects of a sedentary lifestyle have also been established. Every study that included high-impact training has positively impacted BMD, concluding that postmenopausal women should incorporate physical activity into their day-to-day routine. The earlier the exercise regime begins, the less the bone loss, as women lose the most density in early menopause (first one to eight years), and it ensues into a period of lower rates of bone loss. Activities should be continued lifelong to avoid losing previously obtained bone mineralization. If unable to perform high-impact exercises that overload the bone and joints, the secondary option is to enroll in aquatic exercise programs that intertwine high-intensity sequences. As reviewed above, aquatic programs involving bone stimulation with substantial skeletal loading significantly affect BMD and should be considered a great second option. It is necessary to conduct more randomized control trials with larger sample sizes and more extended follow-up periods to expand our knowledge in this research area and form the perfect individualized prescription for preventing osteoporosis and its related fractures.
